# How could metabolomics change pediatric health?

**DOI:** 10.1186/s13052-020-0807-7

**Published:** 2020-03-27

**Authors:** Flaminia Bardanzellu, Vassilios Fanos

**Affiliations:** Neonatal Intensive Care Unit, Department of Surgical Sciences, AOU University of Cagliari, SS 554 km 4,500, 09042 Monserrato, CA Italy

**Keywords:** Neonatology, Obstetrics, Perinatal asphyxia, Breastfeeding, Sepsis, Autism

## Abstract

In the last years, ‘omics’ technologies, and especially metabolomics, emerged as expanding scientific disciplines and promising technologies in the characterization of several pathophysiological processes.

In detail, metabolomics, able to detect in a dynamic way the whole set of molecules of low molecular weight in cells, tissues, organs, and biological fluids, can provide a detailed phenotypic portray, representing a metabolic “snapshot.”

Thanks to its numerous strength points, metabolomics could become a fundamental tool in human health, allowing the exact evaluation of individual metabolic responses to pathophysiological stimuli including drugs, environmental changes, lifestyle, a great number of diseases and other epigenetics factors.

Moreover, if current metabolomics data will be confirmed on larger samples, such technology could become useful in the early diagnosis of diseases, maybe even before the clinical onset, allowing a clinical monitoring of disease progression and helping in performing the best therapeutic approach, potentially predicting the therapy response and avoiding overtreatments. Moreover, the application of metabolomics in nutrition could provide significant information on the best nutrition regimen, optimal infantile growth and even in the characterization and improvement of commercial products’ composition.

These are only some of the fields in which metabolomics was applied, in the perspective of a precision-based, personalized care of human health.

In this review, we discuss the available literature on such topic and provide some evidence regarding clinical application of metabolomics in heart diseases, auditory disturbance, nephrouropathies, adult and pediatric cancer, obstetrics, perinatal conditions like asphyxia, neonatal nutrition, neonatal sepsis and even some neuropsychiatric disorders, including autism.

Our research group has been interested in metabolomics since several years, performing a wide spectrum of experimental and clinical studies, including the first metabolomics analysis of human breast milk. In the future, it is reasonable to predict that the current knowledge could be applied in daily clinical practice, and that sensible metabolomics biomarkers could be easily detected through cheap and accurate sticks, evaluating biofluids at the patient’s bed, improving diagnosis, management and prognosis of sick patients and allowing a personalized medicine. A dream? May be I am a dreamer, but I am not the only one.

## Introduction: the power of metabolomics

In the last years, “Omics” technologies (including genomics, transcriptomics, proteomics and metabolomics) gained popularity and completely modified the scientific approach, due to their role in the description of complexes biological systems, through the simultaneous and usually noninvasive analysis of a large amount of data. Among such tools, metabolomics represents a growing and expanding scientific discipline and a promising area of research.

Metabolomics (also known as Metabonomics), and the word metabolite, share as their root the ancient Greek word, *metabolì*, meaning ‘*change*’.

Metabolomics, an innovative analytical profiling technique, investigates and detects the whole and comprehensive set of molecules of low molecular weight (including sugars, lipids, small peptides, vitamins and amino acids) present in cells, tissues, organs, and biological fluids, portraying their phenotypes.

Thus, the global qualitative and quantitative analysis of all metabolites in biological fluids or tissues is highly useful in human health, allowing the evaluation of specific and individual metabolic responses to many pathophysiological stimuli including drugs, environmental changes, lifestyle, diseases and other epigenetics factors [1].

Metabolomics, detecting broad classes of metabolites, provides a comprehensive functional phenotype integrating clinical features and genetic and non-genetic factors, such as environment, metabolites from symbiotic organisms including gut microflora and xenobiotics [2].

Currently, more than 28.600 articles dealing with metabolomics can be found on PubMed. It is considered a technology that will contribute the world change and, in 2018, 1 Euro out of 18 spent in medicine research were intended for metabolomics.

The human being should be evaluated in a holistic way, and this could be only performed through the integration of genome, transcriptome, proteome, metabolome, microbiome, fungome, virome, bacteriofagome, epigenome, exposome, phenome, diseasome and more.

These topics represents the evolution of scientific technologies applied to medicine and clinical research. In the last decades, intuition-based medicine was replaced by the evidence based- and precision-based medicine. The holistic view of humans as a system biology, the integrate approach and “omics” technologies played a great role in such transition [3].

The main advantages of such technique are a high diagnostic power (detecting changes in metabolites’ concentration and dynamic changes during time), a high rapidity (detecting changes occurring in seconds instead of minutes or hours), and easiness. Moreover, it is a “holistic” (the same metabolite in different samples or tissues at the same time from the same platform) and less expensive science (excluding the initial cost of the instrumentation). On the contrary, the traditional laboratory methodologies offer markers of diseases often showing a low sensitivity or a late appearance.

Metabolic fingerprints can be generated by several analytical techniques, including nuclear magnetic resonance spectrometry (^1^H-NMR), gas chromatography mass spectrometry (GC-MS) and liquid chromatography mass spectrometry (LC-MS), providing metabolic information potentially related to physiological states or pathological conditions of an organism.

In example, variations of these fingerprints determined by changes in concentration of specific metabolites can allow the early detection of the onset of diseases. A strength point of metabolomics is the detection of rapid daily variability in the metabolic fingerprint, representing a metabolic “snapshot.”

The final phenotype is determined by environmental effects, like diet, age, lifestyle, drugs or diseases, on the individual genome, giving origin to a unique transcriptome, proteome and a highly sensible metabolome [4].

Metabolomics is what really happened, since metabolites are the final products of the interactions of genes, RNAs and proteins, while genomics represents what could potentially happen and proteomics is the picture of what is happening. It could be affirmed that metabolomics approach is the equivalent of investigating in a personal dumpster.

Innovative, interesting and promising fields of metabolomics applications are the investigation of physiological status and the diagnosis of a disease, the identification of perturbed pathways due to disease or treatment, the evaluation of the response to drugs (Pharma-metabolomics) and the monitoring of the effects of nutrition (Nutri-metabolomics), the discovery of new and specific biomarkers, the classification of different phenotypes and functional genomics, and, finally, the characterization of natural or artificial products’ composition.

Metabolomics also allows the study of disease phenotypes, molecular pathophysiology and cellular metabolism through metabolic profiling.

Big data, provided by ‘omics’ tools, analyzed through the technology of machine intelligence, have also been named the black box of medicine, providing the so called “artificial intuition”.

Machine techniques can decode the high volume of data generated from a limited number of subjects, comparing them to traditional analytic approaches [5]. From a drop into the ocean (old biochemistry) to an ocean into a drop (metabolomics).

In fact, the modern analytical technologies allow the identification of patterns that confer significantly more information than the measurement of a single parameter, as a bar code contains more information than a single number.

Many authors recently evidenced the strong correlation between metabolomics and clinical research [6, 7].

In metabolomics experiment, the setting, the patients and the types of samples should be carefully chosen. Samples require a correct storage and, after processing, undergo a multivariate statistical analysis whose results are interpreted (Scale free networks) to individuate significant associations or formulate new hypothesis. Samples that can be analyzed in prenatal and perinatal metabolomics analysis are collected from mothers (amniotic fluid, placenta, blood, urine, breast milk, erythrocytes, hair, and vaginal secretions) or from the neonate (urine, blood, saliva, broncho-alveolar fluid, exhaled air condensate, stools, and umbilical cord) [8].

In metabolomics, the complex systems are analyzed using a scale-free topology. Technically, networks expand through the addition of recent vertices, and vertices attach preferentially to well-connected sites. Scale-free networks can be exactly determined using vital features such as a disease or a patient. This allows the comprehension of complex metabolomics systems [9].

Metabolic networks can correlate interlinking metabolites, revealing novel key pathways [10] and the scale free network is useful to identify unexpected pathophysiological mechanisms.

A metabolomics fingerprint (a reflection of the whole metabolome in a biofluid) characterizing a single sample constitutes such a strong characteristic of each subject as to allow its identification with 100% probability. It can be obtained through statistical analysis performed on NMR or GS spectra of multiple samples and points out an invariant part characteristic of each subject. Metabolomics fingerprints can be obtained in a fast, untargeted, and highly reproducible manner, detecting with high sensitivity information regarding the smallest concentration changes of several metabolites at the same time. Thus, an individual metabolic phenotype exists and is the key point of metabolomics clinical studies. However, changes related to pathological stimuli may be difficult to distinguish from physiological variations [1].

In conclusion, metabolomics could help in the optimization of individualized therapy and nutrition; assessing drug-related efficacy or toxicity, identifying phenotype changes associated to disease onset and progression, improving early diagnosis and prognosis [1]. A very interesting and innovative application of metabolomics is sportomics [11]. Metabolomics could improve efficacy precision and accuracy, opening the way to one-person trials, with high efficacy and low toxicity [12].

Currently, a clear and well-defined correlation between each metabolite and the related clinical meaning is not available, even if we are working at the creation of specific atlas dealing with metabolites involvement in several pediatric and neonatal diseases and conditions [13].

## Metabolomics in some organ and pathology involvement

Below, we report only few clinical insights obtained through metabolomics application.

### Heart

Metabolomics seems promising in cardiovascular disease, the leading cause of death and a major cause of disability worldwide including myocardial ischemia, infarction and coronary heart disease. Metabolomics could help in the identification of biomarkers able to early detect risks of such diseases before clinical signs, allowing prevention and early intervention that could prevent fatal consequences [14].

That gut microbiota–host crosstalk seems the gap between cardiovascular risk factors, diet, and cardiovascular residual risk, via translocation through the intestinal barrier [15]. An extensive review on such topic was recently published [16].

Metabolites involved in cardiovascular risk are often related to gut microbiota; i.e., trimethylamine N-oxide (TMAO), has recently been linked to atherosclerosis and thrombosis rate increase. TMAO levels seem to correlate with the risk of cardiovascular events in patients with prior ischemic stroke, via the increase of proinflammatory monocytes [17, 18].

As reviewed by Chalkias and co-workers, metabolomics could be also applied in cardiac arrest, allowing the detection of metabolites linked to cardiac metabolism that could result useful biomarkers in the assessment of an increased risk of cardiac arrest and potentially improving the prevention and treatment of such condition [19].

### Auditory organ

Moreover, in the idiopathic sudden sensorineural hearing loss, a frequent emergency whose aetiology is still unknown, metabolomics seems helpful in the early prediction of clinical outcome and therapy response to steroids (avoiding overtreatment of non-responders).

A recent study evaluated through ^1^H-NMR the urinary metabolome of patients with idiopathic sudden sensorineural hearing loss, and analyzing it according to the clinical outcome after steroids. Among evaluated subjects, a group was composed by healthy controls, a group by patients who did not recover from hearing loss after steroids and finally, patients who recovered after treatment were included. Urinary metabolome resulted significantly different between responders and non-responders, whith B-Alanine, 3-hydroxybutyrate and TMAO higher, and citrate and creatinine lower in the second group [20].

### Kidney

In the field of pediatric nephrourological diseases, in 2010, urinary metabolome (with ^1^H-NMR) between *n* = 21 children affected by nephrouropathies (including renal dysplasia, vesico-ureteral reflux, urinary tract infection, and acute kidney injury), and *n* = 19 healthy children was evaluated. As result, samples belonging to these two groups showed a clear and significant separation. Thus, metabolomics seems a promising, non-invasive tool in nephrourological diseases [21].

Moreover, it seems possible to apply metabolomics in the early prediction of Chronic Kidney Disease in healthy adults born of extremely low birth weight (ELBW) [22].

Some papers were published in this field, investigating metabolomics in pediatric and adult nephrology [23, 24]. Moreover, the role of urinary neneutrophil gelatinase-associated lipocalin (uNGAL) and kidney injury molecule-1 (KIM-1) as predictors of kidney injury severity was studied [25].

### Cancer

Promising results have been also obtained through metabolomics application in oncology [26], even in pediatrics. Such technology could help in disease characterization, monitoring and therapeutic management [27].

Finally, metabolomics can point out markers of hypoxic metabolism in cancer cells [10].

## Metabolomics in obstetrics

Fetal life and perinatal period are crucial phases for neonatal development. The triggers and the conditions to which the fetus is exposed represent essential factors influencing the development of the newborn. In fact, as highlighted by the concept of perinatal programming, when a developing organism is exposed to specific intrauterine conditions, including excessive or inadequate nutrition, puts into practice several adaptive mechanisms and responses potentially modifying its development trajectory; therefore, in such window of vulnerability (or opportunity), persistent short- and long-term effects on newborn phenotype are performed.

Thus, intrauterine stimuli and factors that are present during the early perinatal life can affect fetal and neonatal development and lead to negative consequences [28–30].

In the evaluation of pre- and peri-conceptional factors, preterm birth and labor, intrauterine growth restriction (IUGR), maternal gestational diabetes (GDM), pre-eclampsia (PE), fetal infections, exposition to hyperoxia during post-natal life, potentially related to epigenetic changes on DNA [31], metabolomics application could give great advantages, both in the comprehension, diagnosis and treatment of such conditions and represents a promising field to investigate [32].

The early diagnosis of the prediction of several maternal complications represents a challenge due to the high complexity of these conditions, still partially understood [32].

Moreover, recent evidence highlights the pivotal role played by the placenta during fetal life. Such structure can be defined a metabolic interface between the mother and the fetus, influencing neonatal maturation and metabolism [33].

Several metabolomics studies are currently available on common pregnancy complications. Step forwards, in the field of obstetrics, could improve pregnancy management and delivery assistance, with positive effects on maternal and fetal health, the reduction of costs, cesarean sections and hospitalization.

Thus, we report some examples of metabolomics application in Human cytomegalovirus (HCMV) congenital infection, maternal obesity and preterm labor.

HCMV, a potentially fatal viral infection during pregnancy, seems to alter metabolic profile of amniotic fluid (AF), in relation to maternal and fetal response to such infection.

As evidenced, through GC-MS analysis of AF, different profiles characterize pregnant women that transmitted HMCV infection to their fetuses (*n* = 20), instead of mothers who acquired but not transmitted the virus (*n* = 20) and healthy controls (*n* = 23). Moreover, AF samples from mothers whose neonates resulted symptomatic at birth (*n* = 9) were clearly separated from AF belonging to neonates who acquired HCMV infection but did not show clinical signs, especially regarding metabolites related to fatty acids biosynthesis [34].

In conclusion, metabolomics could describe maternal and fetal status in congenital HCMV infection, allowing an early diagnosis and an accurate management [34], and, in our opinion, it could be also applied in the evaluation of breast milk acquired CMV infection [35].

In the field of maternal obesity (predisposing to pregnancies and fetal complications and potentially impairing neonatal outcome), we performed the first metabolomics study evaluating placental samples in normal weight (*n* = 20) and obese (*n* = 18) pregnant women, often affected by GDM as comorbidity.

Through GC-MS, significant differences in antioxidant metabolites, nucleotide production, lipid synthesis and energy production were detected. In detail, obese mothers’ placentas also showed a peculiar fatty acids profile, related to an increase in placental metabolism and potentially reflecting intrauterine changes responsible of later diseases, including metabolic syndrome and cardiovascular disease [33].

Metabolic pattern linked to the onset of labor, potentially detected by metabolomics tool, were pointed out in another study. Urine samples (*n* = 59) were collected from a group of full-term pregnant women before and after the onset of labor and their metabolic discriminating features were pointed out through GC-MS and ^1^H-NMR. As result, 18 metabolites allowed the discrimination between urinary samples of women in labor and not in labor (NL).

In detail, glycine, alanine, acetone, 3-hydroxybutiyric acid, 2,3,4-trihydroxybutyric acid and succinic acid, characterized the late phase of labor. Thus, metabolomics could also help in discriminating urine samples from women in labor, offering a precocious screening of the onset of such condition [36].

Another obstetric complication is represented by the premature rupture of membranes (PROM), defined as the fetal membranes’ rupture prior than the onset of labor. It can determine an increased rate of infections (such as chorioamnionitis and endometritis) and other complications for the mother and the fetus itself (abnormal fetal presentation, neonatal sepsis, intra-ventricular hemorrhage). PROM can occur at any gestational age (GA) and is often related to premature birth.

Thus, PROM diagnosis should be promptly recognized and early and adequately managed (avoiding unnecessary antibiotics), reducing potential risks. However, sensible biomarkers are still lacking. Urinary maternal metabolome with CG-MS, describing interesting pathways associated with PROM and preterm labor, was therefore investigated.

A total of *n* = 38 urinary samples was collected out of a group of *n* = 38 full-term pregnant women, divided into three subgroups: In the first, *n* = 11 women without PROM and labor, in the second, *n* = 10 pregnant women with PROM without labor, in the third, *n* = 17 pregnant women with PROM and labor were included.

As result, the reduction of 9 metabolites resulted significantly associated with PROM (galactose, uric acid, 3,4-dihydroxybutyric acid, galactitol, alanine, lysine, 4-hydroxyphenylacetic acid, serine, and hydroxyproline dipeptide).

Moreover, 60 metabolites significantly varied between the second and the third group. Most of them were higher in the group with PROM and labor, while phosphate, lactose, and uric acid were higher in the group with PROM without labor; among the increased metabolites in this group, 3,4- dihydroxybutyric acid is an intermediate of fatty acids oxidation (increasing in infections to provide energy), glucuronic, gulonic, glucaric, and gluconic acids are related to oxidative stress, cis-Aconitic acid (intermediate of tricarboxylic acid cycle), faces the increase in energy demand [37].

Regarding spontaneous preterm birth, a still partially characterized cause of neonatal mortality and short- and long-term morbidity, metabolomics seems promising in the identification of sensible biomarkers [38].

Potential long-term effects of preterm birth were also evaluated through a metabolomics study in adult patients (mean age 24 years), in which different metabolic urinary profiles were observed in subjects who were born full-term if compared to those showing ELBW [39].

Recently, in a preliminary investigation, a different urinary metabolome was detected in relation to birth modality, in a sample collected in the first hours of life, underlining how this factor could influence neonatal metabolism, organogenesis and determine long-term effects. Full-term neonates born by vaginal delivery, if compared with neonates born by cesarean section, showed higher levels of dicarboxylic acids and Krebs cycle-related metabolites in neonates, probably due to differences in fatty acid oxidation, thermoregulation at birth or energy metabolism. Moreover, bacterial-related metabolites also showed some variations, in relation to a different microbiota colonization according to delivery mode [40].

Finally, also in research in the field of the great obstetrical syndromes (PE, GDM, and IUGR) metabolomics could give a great contribution, improving prevention, early diagnosis, and monitoring, as reported by many authors [41, 42].

PE, a hypertensive gestational disorder originating in the placenta and affecting about 5 to 7% of the pregnancies, can lead to several fetal or maternal complications. It can occur since the 20th week of pregnancy. PE is the association of hypertension, proteinuria and edema, contributing to placental impairment and fetal distress. Related effects can also impair long-term neonatal outcome, potentially influencing his metabolism until adulthood. Due to these reason, the early identification of women at risk of developing PE would be desirable [40] and the biomarkers currently employed in risk prediction are weak outcome predictors.

Among the metabolomics studies in PE, we report few interesting and promising results. Sander and colleagues detected significant changes in serum metabolome (third trimester) of PE pregnant women (*n* = 32), versus healthy controls (*n* = 5), and most varying metabolites were hydroxyhexacosanoic acid, diacylglycerols, glycerophosphoinositols, nicotinamide adenine dinucleotide metabolites, bile acids and products of amino acid metabolism [43].

Moreover, the group of Liu, analyzed the eicosanoid content in serum of PE (*n* = 10) and healthy pregnant women (*n* = 10) through LC-MS; as result, levels of arachidonic acid metabolites and some of the lipoxygenase metabolites of eicosapentaenoic acid (EPA) and docosahexaenoic acid (DHA), were increased, while cytochrome P450 metabolites of EPA and DHA were decreased in women with PE. The values of lleukotriene B4, 14,15-dihydroxy-eicosatetraenoate, 16-hydroxydocosahexaenoic acid and 8,9-epoxy eicosatetraenoic acid resulted significant markers of PE occurrence and progression [44].

According to another study, metabolomics could help in discriminating PE pregnancies associated to pre-term and full-term delivery, evaluating seriate serum maternal samples (12, 20, 28 and 36 weeks of GA) in women with PE delivering full-term (*n* = 165) and pre-term (*n* = 29). Among the obtained results, it emerged that 4-hydroxyglutamate could represent a novel first-trimester predictor of pre-term disease [45].

According to another study, metabolomics placental profiles could also be useful in identifying PE with placental dysfunction and even associated IUGR [46].

Recent metabolomics results in IUGR [5, 47], even in relation to gut dysbiosis [48] and evaluating neonatal urines [49, 50], and in GDM [51–53] are currently available in literature.

In the future, the study of great obstetrical syndromes, old data, could be better performed through the use of new eyes, especially metabolomics. Research should be focused on the interactions between genomics, dysbiosis, environmental factors, including diet, hypertensive and inflammatory factors.

In conclusion, a metabolomic approach in different areas of maternal and perinatal medicine could really help in a best characterization of such conditions and in the identification of novel biomarkers, even if the current findings still require further validation on larger cohorts [32].

The most recent metabolomics studies on amniotic fluids were recently reviewed, describing metabolic interactions between the mother and the fetus in several pathophysiological conditions, highlighting how such technique can describe feto-placental metabolism [54].

## The role of metabolomics in perinatal asphyxia

The potential role of metabolomics in perinatal asphyxia is an intriguing topic, well describing the concepts of great inter-individual variability and the needs of personalized approaches.

Perinatal asphyxia is one of the most frequent causes of neonatal death or impaired outcome, potentially leading to severe disability, cerebral palsy and poor neurodevelopment [55]. Such topic was deeply investigated across several studies, speculating the potential occurrence of specific metabolic perturbations in the urine of asphyxiated newborns. In the first of these studies, urinary samples were collected and evaluated from *n* = 3 full-term male asphyxiated neonates. Those patients, although characterized by the same clinical picture of early seizures and laboratory features (Ph 6.8 and EB – 22 mEq/l), underwent three really different outcomes. One out of three died within 48 h of life, one underwent acute renal and hepatic failure and cerebral palsy at discharge, the last completely recovered and was discharged in good clinical conditions. Thus, the same treatment (hypothermia) could not result the optimal treatment approach in all the asphyxiated neonates, due to the great differences characterizing each individual.

The urinary metabolome of these neonates were analyzed at birth and at the end of hypotermia, with ^1^H-NMR and resulted significantly different, reflecting the three different neonatal outcomes. In detail, mediators such as glycine, valine, maleic acid, and sorbitol varied in relation to asphyxia, while glucose, aspartic acid, asparagine, ornithine, gluconic acid and L-lysine were mostly influenced by kidney damage [23].

By another study, evaluating *n* = 14 neonates (*n* = 6 cases, *n* = 8 controls) through the same platform, we demonstrated that asphyxia-related metabolic urinary variations were also evident 48 h after birth. Hypoxaemia and acidosis mostly determined variations in metabolites involved in energy demand, kidney damage and oxidative stress (lactate, glucose, TMAO, threonine, 3-hydroxysovalerate) [56].

Finally, progressive modifications of urinary metabolome from *n* = 12 asphyxiated newborns were pointed out, compatible with the progression of clinical condition. Urinary samples were evaluated at birth, during and at the end of hypothermia, at a week and a month after birth through GC-MS [57] and ^1^H-NMR [55]. In detail, taurine, hypotaurine, U1710, lactic acid, lysine, mannitol and ubiquinone showed the greatest variations [57].

The urinary metabolome of survivors was clearly different from that of neonates that died in the first 8 days of life, and these differences were already present at birth. These modifies could indicate irreversible asphyxia-related perturbations, indicated by metabolic fingerprints, such as lactic acid, taurine and other metabolites [55, 57].

Moreover, energy deficiency, variations in the cycle of tricarboxylic acids (TCA) and the increase in lactic acid represent negative predictors, while their decrease during post-natal time may represent indicators of aerobic metabolism and homeostasis restoring in surviving infants with the best outcomes [57].

Finally, lactic acid, myo-inositol, betaine increased while citrate, α-ketoglutarate, succinate, acetone, dimethylamine, glutamine, pyruvate, arginine and acetate decreased in asphyxiated newborns’ urinary profiles at 1 month of life [55]. These data highlight that determining a real threshold for survivals characterizing each metabolite could be highly useful.

In a pig model of perinatal asphyxia, urinary metabolomics was also applied to investigate the effect of different oxygen concentration (18, 21, 40, and 100%) administered during resuscitation. In such study, 21% of oxygen resulted associated to the best outcome and metabolic effect, highlighting that metabolomics could also help in monitoring the effects of therapeutic approaches and oxygen supplementation [58].

These findings point out that each patient is characterized by a high inter-individual variability; maybe some patients die or develop side effects due to an overtreatment while others would benefit from a more aggressive treatment approach.

In fact, the current medicine is based on detailed protocols, calibrated on the mean of patients. However, research is revealing that the mean of patients does not exist and the development of precision medicine could be beneficial for each single individual.

If these results were confirmed, metabolomics could help in identifying early markers of perinatal asphyxia, describing the evolution of such condition over time and resulting highly related to neonatal prognosis.

## Metabolomics in neonatal nutrition

Neonatal nutrition is a main relevance topic, being one of the most important factor influencing the early newborns development and affecting short- and long-term outcome, due to the power of breast milk (BM)-associated perinatal programming. In fact, in the first weeks of life, BM is able to change the fate of newborns’ metabolism [59].

BM contains water (88%) nutrients (lipids, carbohydrates, proteins, vitamins, minerals), bioactive components as growth-factors (GFs), hormones, cytokines, chemokines, antimicrobial compounds like immunoglobulins (Ig), a specific microbiome and BM-related cells including epithelial, immune cells and multipotents stem cells (SCs) [60–62].

BM represents the ideal biofluid for neonatal nutrition, especially if premature, able to modify its composition according neonatal needs, especially in terms of GA or lactation stage [63, 64].

Among BM components, BM oligosaccharides (HMO) can shape neonatal gut microbiome, influencing immune system development, protecting against infections and reducing necrotizing enterocolitis (NEC) rate [65–67].

HMOs composition in BM highly depends on maternal genetic factors, since mothers can be divided in Secretors (*Se+*) and non Sectetors (*Se-*) according to the expression of the enzyme α-1-2-fucosyltransferase (FUT2), codified by *Se* gene. Metabolomics studies evidenced a clear separation between BM of *Se +* and *Se-* mothers, with a higher content of fucosylated oligosaccharides, 2α-fucosyl-lactose, lacto-difucotetraose, lacto-N-fucopentaose and lacto-N-difucoesaose in the first group. Thus, neonates of *Se-* mothers could benefit from specific HMOs supplementation, to avoid NEC and other infections [68].

Even BM microbes, also called maternal “lactobiome” seems to influence neonatal outcome [69].

Thus, understanding BM composition and its effects is a central issue of modern research. A personalized nutrition based on the features of each newborns would be desirable. In this perspective, metabolomics represents an ideal tool to analyze BM and the composition of different types of commercial available formula milks (FM), allowing their improvement to resemble as possible BM composition.

Moreover, metabolomics results also promising in the detection of drugs and contaminants in BM, helping in the determinations of its safety in specific conditions or maternal exposition of environmental toxicants [70, 71].

The first metabolomic study evaluating BM composition was performed in 2012 by our research group. BM from *n* = 20 mothers delivering neonates of GA 26–36 weeks, and from *n* = 3 full-term delivering mothers was collected from 1 to 13 weeks post-partum. In the same study, samples of FM were also analyzed. As result, a clear separation occurred in BM vs FM (samples analyzed through ^1^H-NMR and GC-MS). In fact, a higher lactose concentration was found in BM, while maltose resulted higher in FM. Some differences also characterized FAs profile, such as oleic and linoleic acids that were higher in FM.

Although on a small number of samples, a correlation with GA was highlighted, especially regarding carbohydrates; i.e., lactose increased during milk maturation [63].

Interesting results emerged from the analysis of metabolic effects of different nutrition regimens on neonates. The urinary metabolome in the first week of life in three groups of newborns (GC-MS), divided in appropriate for gestational age (AGA), small for gestational age (SGA) and large for gestational age (LGA) was compared. This study highlighted the power of early nutrition on neonatal metabolic pathways. In fact, despite a clear separation showed by urinary metabolome of AGA group than LGA and SGA at birth, at 1 week of life, urinary samples were mostly influenced by nutrition, with a clear separation between the samples of breastfed neonates and those exclusively fed with FM [59].

It was also demonstrated that such significant nutrition-related metabolic differences can persist up to 4 months of life. The metabolites showing the greatest variations (measured through 1H-NMR) were those related to energy metabolism, antioxidant action, neuro-modulation and brain development, surfactant synthesis; moreover, variations were detected in HMOs content and intestinal microbiome-produced metabolites. These findings highlight the effects of early nutrition on neonatal development [72].

Recently, a unique study on BM collected from mothers delivering preterm multiples (*n* = 19 couples and *n* = 5 triplets) from birth and up to 20th w of postnatal life pointed out a greater protein content in BM of preterm multiples than singletons matched for GA, despite a lower content of lactose. The higher protein content in BM for preterm multiples could face the nutritional and development needs of such vulnerable category [73].

In a preliminary investigation comparing metabolomics profiles of *n* = 15 different kinds of FM including *n* = 6 organic –bio formulas vs BM, a significantly higher methionine content in organic bio-formulas (about 3 folds higher) than conventional FM was pointed out [74]. This result is highly interesting taking into account that methionine is an epigenetic mediator, involved in methylation.

In pathological conditions, BM could interfere with neonatal development and can impair the function of several organs [75].

In this regard, we recently focused on Great Obstetrical Syndromes-GOS (PE, GDM and IUGR), pointing out that BM metabolome could be altered in such conditions, through unknown mechanisms including maternal deficiency of specific metabolites and potentially involving inflammatory triggers. Such altered BM composition will affect neonatal development, contributing to the adverse long-term outcomes in children born by mothers affected by GOS and therefore exposed to an altered intrauterine environment [75].

In a rat model, it has been also demonstrated that maternal obesity and non-alcoholic fatty liver disease (NAFLD) influence offspring metabolism, predisposing to dysmetabolism, insulin resistance, obesity and NAFLD itself, potentially via BM (in fact, a higher content of leptin was detected in these mothers instead of healthy controls) [76].

Finally, BM contains several populations of cells, including epithelial cells, immune cells and stem cells (SCs) named human breast-milk derived SCs (BMSCs) [77–83].

These cells take part in mammary gland proliferation during pregnancy and lactation, end express different and specific markers according to lactation stage [84–86] and GA at birth [87, 88].

The greatest potential of BMDSCs on neonatal outcome depends on the ability, after neonatal ingestion, to pass through neonatal gut into circulation, where they could survive and be transferred into brain and other neonatal organs, influencing their development [89, 90].

Since BMDSCs resulted able to differentiate into several cellular lines, including nervous cells and neural SCs [79, 91], the transfer of BMDSCs through breastfeeding could improve the maturation of neonatal brain and other organs, especially in premature neonates [86, 92, 93].

## Metabolomics and sepsis

Sepsis, potentially caused by viruses, bacteria or fungi, is a frequent cause of neonatal morbidity and mortality, especially if affecting the highly susceptible premature newborns. “Early onset” sepsis (EOS) occurs within 72 h of life, while “late onset” sepsis (LOS) between 72 h and 6 days of life. Although sepsis represents a life-threatening condition, current biomarkers lack in diagnostic accuracy. The early and sensible diagnosis of such condition, based on reliable and accurate mediators, could improve its management and prognosis. Currently, sepsis diagnosis, allowed by blood microbiological culture, is often delayed [94].

Thus, metabolomics approach could provide new chances in the diagnosis of sepsis, in clarifying its pathogenic mechanisms and prognosis, through the definition of novel sensible biomarkers, as reviewed by Lee and colleagues [95].

Metabolomics could reveal sepsis-related metabolic pathways, such as hypoxia, oxidative stress, and increased energy needs (influencing glucose levels and oxidative metabolism of fatty acids) [94, 96].

Moreover, preventive and therapeutic strategies could benefit from the whole comprehension of the interactions among the host and its microbiome, playing a pivotal role in sepsis progression [95].

Some studies available in literature, briefly reviewed above, show promising result, even if they require further confirmation on larger samples.

In the first of them, urinary samples collected at a single time-point from septic neonates (including both EOS and LOS) was compared to healthy controls (^1^H-NMR and GC-MS), highlighting effects on energy metabolites (including the increase in glucose, maltose, lactate, acetate, ketone bodies intermediates, and effects on antioxidants) [97].

Moreover, in another study, a different urinary profile in a preterm neonate affected by fungal sepsis instead of healthy controls (GC-MS) was pointed out. Among the observed results, some proteolysis-related amino acids suggesting a hypermetabolic and hypercatabolic state increased, and the metabolite D-serine resulted a good predictor of antifungal treatment response, reducing itself during therapy [98].

In a study of Serafidis and co-workers, urinary metabolic profile evaluated through ^1^H-NMR and LC-MS allowed a clear discrimination between septic and non-septic neonates, analyzing samples collected at the time of diagnosis, and after 3 and 10 days. Metabolite variations disappeared at the end of the symptoms, giving promising information for prognosis and therapy [99].

The group of Stewart and co-workers demonstrated that metabolomics application could also give interesting results on fecal samples; in fact, they showed the role of gut microbiome is involved in the pathogenesis of LOS. By comparing a group of healthy neonates to LOS affected infants, Bifidobacteria (with protective beneficial effects) resulted higher in the first group, in conjunction with prebiotic oligosaccharides, raffinose, sucrose, and acetic acid. Interestingly, the same bacterial species isolated by blood culture were dominant in gut microbial community, as consequence of a bacterial translocation from the gut into circulation and arguing for the first time a dependence of neonatal sepsis on gut dysbiosis [100].

Finally, metabolomics was also applied in pediatric sepsis, in a cohort of *n* = 60 septic pediatric patients (including *n* = 7 newborns); their serum metabolome was compared with *n* = 40 healthy pediatric controls and showed an increased in lactate, glucose, creatinine, 2-oxoisocaproate, 2-hydroxysovalerate and 2-hydroxybutyrate and lower levels of threonine, acetate, 2-aminobutyrate, and adipate [101].

In conclusion, metabolomics could provide precocious and accurate sepsis marker in the perspective of an early diagnosis and a tailored management, potentially reflecting disease progression, therapy related efficacy, and toxicity [98, 102–104].

## Metabolomics and neuropsychiatric disorders

The expression autism spectrum disorders (ASD) includes a group of neurodevelopmental disorders characterized by delayed or impaired language development and difficulties in social interactions, and repetitive and stereotyped behaviors. The pathogenesis of such condition, showing a high heterogeneity, includes the interaction among genetic factors, environmental risk factors, socioeconomic status, maternal and neonatal infections, prenatal nutrients, immune deregulation, maternal exposure to potentially toxic drugs, formula feeding, and epigenetic components (including DNA methylation), even if the exact mechanisms are not fully understood, up to now. Despite ASD rate rapid increase in recent years, their diagnosis is still largely based on clinic signs and sensible biomarkers are not available. Metabolomics recently emerged as promising tool for a better characterization of such diseases, allowing the individuation of sensible biomarkers, their monitoring and maybe the introduction of innovative treatments.

In particular, the metabolites mostly associated to ASD seems those involved in amino acid metabolism, cholesterol metabolism, folate abnormalities, antioxidant status, nicotinic acid metabolism, and mitochondrial metabolism. A great role could also be played by some metabolites derived from the gut microbiota, potentially shaping ADS children behavior, metabolic patterns and immune response [105–108], tryptophan, vitamin B6, purine metabolic pathways, phenylalanine and tyrosine biosynthesis, intermediary compounds of the TCA cycle [108].

Metabolic features of autistic children were evaluated in several studies; some of them are reviewed above.

By two recent studies, the involvement of oxidative mechanisms and intestinal microbiome in ASD predisposition was detected, via the interaction with the gut-brain axis and to the lack of the intestinal mucosal barrier.

Through the analysis of urinary samples from *n* = 21 ASD versus their *n* = 21 healthy siblings (aged between 4 and 17 years), different levels of oxidative metabolites, carbohydrate metabolism intermediates, bacterial-derived metabolites suggesting an increase in *Clostridia spp*. in the gut were detected. Moreover, aromatic amino acids precursors of neurotransmitters and key hormones for the nervous system such as catecholamine and serotonin also showed variations. These results evidence that diet is a relevant epigenetic factor, influencing gut microbiome, in ASD pathogenesis [109, 110].

In ASD children, the increase in *Clostridium, Alistipes, Akkermansia, Caloramator, Sarcina spp*., and the reduction in *Prevotella spp., E. siraeum,* and *Bifidobacterium spp. occurred, with consequent alterations in urinary levels of* hippuric acid, p-hydroxyphenylacetic acid and 3-(3-hydroxyphenyl)-3-hydroxypropanoic acid, and propionic acid [108].

Plasma metabolome of ASD children was also evaluated by Orozco and colleagues, who described interesting metabolic alterations related to the impairment in neurodevelopment, also taking into account the potential overlap between ASD and other causes of developmental delay, including Down Syndrome and idiopathic-developmental delay [111].

Moreover, since ASD prevalence is higher in males, metabolomics approach was also applied to investigate the urinary pathways in males and female patients, analyzing the possible molecular causes of such gender difference and trying to find sensible biomarkers for the diagnosis of ASD potentially related to the subject’s gender. In detail, the authors evidenced a significant increase in the levels of adenine, 2-methylguanosine, creatinine, 7alpha-hydroxytestololactone and a decrease in creatine in females; thus, they identified creatinine:creatine ratio as potential marker of ASD in females [112].

Metabolomics perturbations associated to ASD cannot be only detected in blood, urine or saliva, but even in cerebellum or cortex samples of affected patients. In the study of Kurochin et al., 1366 metabolites were compared in the prefrontal cortex grey matter of ASD patients and healthy controls, revealing different profiles and metabolic pathways and opening the way to different analysis strategies [113].

Finally, the potential pathogenic effects of some drugs (including thalidomide and, as recently highlighted, acetaminophen) on ASD development should be further investigated [108].

A clinical promising application of metabolomics in neuropsychiatric disorders was recently reviewed. The current evidence on Pediatric Acute-onset Neuropsychiatric Disorder (PANS), a clinical condition characterized by sudden obsessive-compulsive symptoms and a close dependence on infective triggers and potential post-infectious immune-mediated mechanisms, was analyzed. In this regard, a metabolomics approach was applied to investigate the case of a 10 year-old girl with PANS. Our evaluation of her urinary metabolome was performed before and after the treatment with clarithromycin, macrolides playing antimicrobial activity and potentially acting as immunomodulatory agent. During such pharmacological treatment, a great improvement of her clinical manifestations with the reduction of symptoms was observed, in addition to a clear modification in urinary metabolomics pathways, especially regarding metabolites related to protein biosynthesis, energy metabolisms, aminoacids involved in brain functions and microbial products also related gut colonization [114].

Metabolomics seems also promising in providing metabolic information improving early recognition and potentially modifying the treatment and prognosis of inborn errors of metabolism (IEMs), as recently reviewed. Through a new approach based on the integration of metabolomics and genomic data, a set of metabolites selectively influenced by a specific gene inactivation in urine, blood or other biofluids could improve our knowledge of disease-related metabolites in various genetic condition [115].

The study and application of metabolomics to neuropsychiatric disorders seems to be a promising tool for the immediate future, potentially identifying specific biomarkers, involved since the early phases of fetal life and influencing neurodevelopment from perinatal programming to adulthood [116–118].

## Conclusions

Metabolomics has been extensively studied in Medicine, as evidenced by the presence of more than 28.600 related papers on PubMed, until now. In the last years, many articles and reviews have been published in the fields of obstetrics, perinatology, neonatology and pediatrics.

Metabolomics resulted a highly promising tool in the early diagnosis of several fetal, perinatal, pediatric and adulthood conditions, through the detection of specific and sensible biomarkers. Moreover, metabolomics could help in monitoring the disease progression, in optimizing therapy and in the evaluation of related side effects, in the perspective of a tailored management.

In the next years, we will move towards a personalized holistic approach in pediatrics. Even if none of the mentioned techniques was yet validated for current clinical application, it is reasonable to predict that available results could be confirmed on larger cohorts of patients and through a more close standardization of protocols and studies.

In the future, we hope that metabolomics sensible sticks could be included in clinical practice, potentially accurate and cheap, to investigate rapidly neonatal biofluids and to introduce an innovative and personalized therapeutic management at the patient’s bed.

However, it is possible to predict that metabolomics will change pediatrics in the immediate future, representing both an evolution and a revolution. We could say that metabolomics can bring from small metabolites to big ideas, and we hope it could provide more chances to sick neonates and children, highlighting what is good health and revealing how to maintain and defend it, improving well-being and preventing diseases. A dream? May be I am a dreamer, but I am not the only one.

## Data Availability

Not applicable.
